# Computational Fluid Dynamic Simulations of Maternal Circulation: Wall Shear Stress in the Human Placenta and Its Biological Implications

**DOI:** 10.1371/journal.pone.0147262

**Published:** 2016-01-27

**Authors:** E. Lecarpentier, M. Bhatt, G. I. Bertin, B. Deloison, L. J. Salomon, P. Deloron, T. Fournier, A. I. Barakat, V. Tsatsaris

**Affiliations:** 1 INSERM, UMR-S 1139, Paris, France; 2 PRES Sorbonne Paris Cité, Université Paris Descartes, Paris, France; 3 Port Royal Maternity, Department of Gynecology Obstetrics I, Centre Hospitalier Universitaire Cochin Broca Hôtel Dieu, Groupe Hospitalier Universitaire Ouest, Assistance Publique-Hôpital de Paris, Paris, France; 4 DHU Risques et grossesse, Paris, France; 5 PremUP Foundation, Paris, France; 6 Institut de Recherche pour le Développement (IRD), MERIT - UMR216, Paris, France; 7 Laboratoire d'Hydrodynamique (LadHyX), CNRS, École Polytechnique, 91128, Palaiseau, France; 8 INSERM U970, Paris Cardiovascular Research Center-PARCC, Paris, France; 9 EA FETUS 7328, Paris, France; 10 Department of Obstetrics and Fetal Medicine, Paris Descartes University, Hôpital Necker-Enfants-Malades, Assistance Publique-Hôpital de Paris, Paris, France; University of Washington, UNITED STATES

## Abstract

**Introduction:**

In the human placenta the maternal blood circulates in the intervillous space (IVS). The syncytiotrophoblast (STB) is in direct contact with maternal blood. The wall shear stress (WSS) exerted by the maternal blood flow on the STB has not been evaluated. Our objective was to determine the physiological WSS exerted on the surface of the STB during the third trimester of pregnancy.

**Material and Methods:**

To gain insight into the shear stress levels that the STB is expected to experience *in vivo*, we have formulated three different computational models of varying levels of complexity that reflect different physical representations of the IVS. Computations of the flow fields in all models were performed using the CFD module of the finite element code COMSOL Multiphysics 4.4. The mean velocity of maternal blood in the IVS during the third trimester was measured *in vivo* with dynamic MRI (0.94±0.14 mm.s^-1^). To investigate if the *in silico* results are consistent with physiological observations, we studied the cytoadhesion of human parasitized (*Plasmodium falciparum*) erythrocytes to primary human STB cultures, in flow conditions with different WSS values.

**Results:**

The WSS applied to the STB is highly heterogeneous in the IVS. The estimated average values are relatively low (0.5±0.2 to 2.3±1.1 dyn.cm^-2^). The increase of WSS from 0.15 to 5 dyn.cm^-2^ was associated with a significant decrease of infected erythrocyte cytoadhesion. No cytoadhesion of infected erythrocytes was observed above 5 dyn.cm^-2^ applied for one hour.

**Conclusion:**

Our study provides for the first time a WSS estimation in the maternal placental circulation. In spite of high maternal blood flow rates, the average WSS applied at the surface of the chorionic villi is low (<5 dyn.cm^-2^). These results provide the basis for future physiologically-relevant *in vitro* studies of the biological effects of WSS on the STB.

## Introduction

Pregnancy causes major physiological changes in the mother’s circulation. The maternal cardiac output rises by 30 to 35% and the total uteroplacental blood flow increases to about 25% of the total cardiac output. The chorionic villus is the structural and functional unit of the human placenta. By the end of the first trimester of pregnancy, the maternal blood, carried by the spiral arteries, circulates between the chorionic villi in the intervillous space (IVS) of the placenta. The villi are delimited by a double layer of epithelial cells: the mononucleated villous cytotrophoblasts (CTs) fuse to form the syncytiotrophoblast (STB) surrounding a mesenchymal core containing fetal vessels. The STB is in direct contact with maternal blood [1] whose viscous flow generates a wall shear stress (WSS) (i.e. frictional force per unit area) that acts tangentially to the apical membrane of the STB.

It is now acknowledged that endothelial cells respond to WSS by changing their morphology, function, and gene expression [2]. Endothelial responses to WSS play a critical role in blood flow—dependent processes, including vasodilation [3], angiogenesis, vascular remodeling, and atherosclerosis [4]. Although the STB is usually likened to an endothelium, the biological effects of WSS on the human STB have not been studied. The aim of the present study was to determine the mean WSS due to maternal blood flow on the STB during the third trimester of pregnancy. We therefore developed physiologically realistic computational models of the IVS of a normal placenta in the third trimester of pregnancy. We subsequently performed computational fluid dynamic simulations of maternal blood flow in these models to determine the WSS exerted on the STB.

## Methods

### Ethics Statement

The local ethics committee (Comité de Protection des Personnes Ile de France III) approved the PLACENTIMAGE study and the human primary cell culture studies. All women provided written informed consent for participation in these studies.

### Computational Modeling of the Intervillous Space

To gain insight into the shear stress levels that the STB is expected to experience *in vivo*, we have formulated three different computational models of varying levels of complexity that reflect different physical representations of the IVS. These three models are described next.

### Model 1: Two-dimensional model of the intervillous space as a porous medium

**Fig 1:**
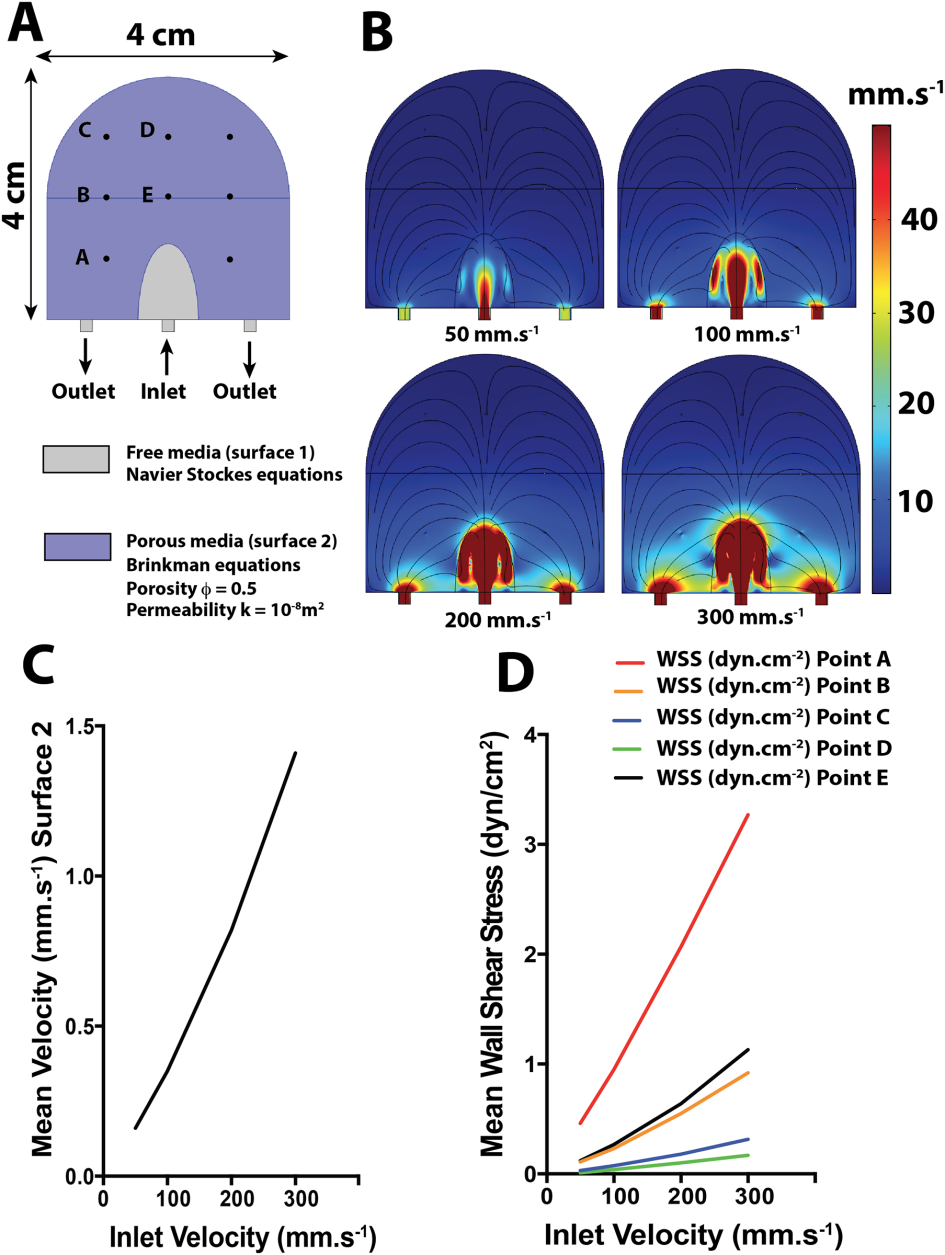
2D modeling of a human placentone as a porous medium and simulation of maternal circulation in the IVS. A: Modeling of the placentone as a porous medium (porosity ϕ = 0.5, permeability k = 10^−8^). The placentone contains a central inlet 2 mm in diameter (spiral artery), two identical 2 mm-diameter outlets (decidual veins) and a central cavity above the spiral artery. Eight circles with the same diameter as a terminal villus (50 μm) are symmetrically distributed in the placentone. The wall shear stress applied to the perimeter of 5 circles (A, B, C, D, E) was computed. B: Velocity plots for the 4 inlet velocities (50, 100, 200 and 300 mm.s^-1^). C: Mean fluid velocities (mm.s^-1^) in the porous medium as a function of the inlet velocity. D: Mean wall shear stress (WSS) (dyn.cm^-2^) applied to the contour of each of the 5 circles (A,B,C,D,E) as a function of inlet velocity.

In this initial model, the functional placental unit (placentone) is assumed to be enclosed by an impermeable 4 cm-high and 4 cm-wide border having the shape shown in Fig 1A. At the base, there is a 2 mm-wide central flow inlet that represents the spiral artery as well as two identical 2 mm-wide side outlets that represent decidual veins (Fig 1A). The villous tree in the placentone is modeled as a porous medium of uniform and isotropic permeability (k = 10^−8^ m^2^) within which flow is governed by the Brinkman equations. The placentone also contains a central cavity above the spiral artery in order to match physiological observations [5]. Flow in this cavity is governed by the Navier-Stokes equations. No slip boundary conditions are applied to the entire solid contour, and continuity of both velocity and stress is assumed at the interface between the central cavity and the porous medium. As shown in Fig 1A, five circular regions with the diameter of a typical terminal villus (50 μm) [5] are defined in the porous medium. The average WSS on the perimeters of five of these circles (denoted as A-E in Fig 1A) was calculated to provide representative values within the villous tree.

### Model 2: Two-dimensional modeling of the intervillous space based on anatomic images

**Fig 2:**
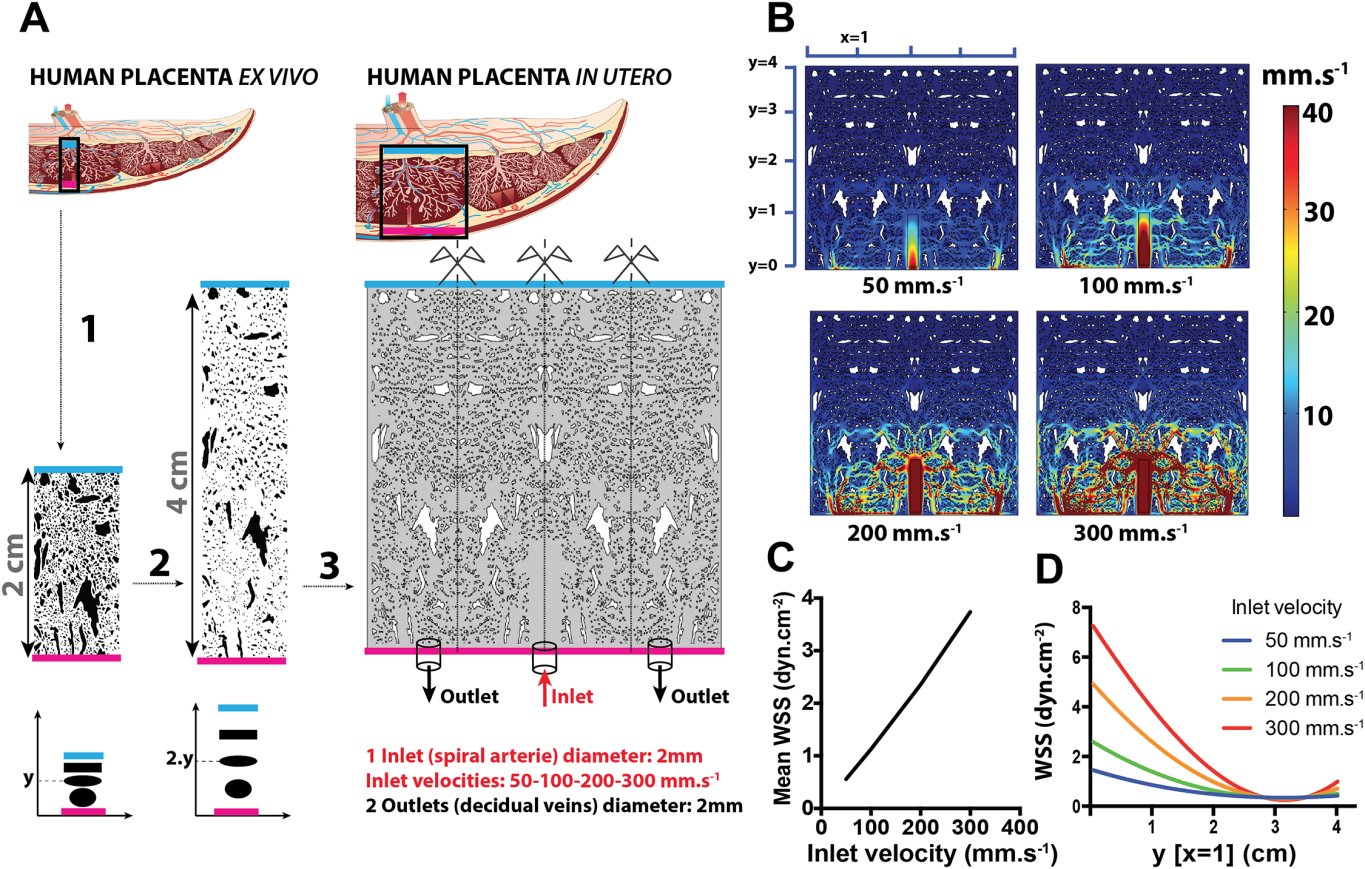
2D realistic modeling of a human placentone and simulation of maternal circulation in the IVS. A: (1) Digitization, binarization and vectorization of a vertical cross-section from the chorionic plate (blue line = fetal side) to the basal plate (pink line = maternal side) of a third-trimester normal human placenta. (2) Correction of the placental collapse; the coordinates (x_A_;y_A_) of the center of gravity for each solid element were determined, and the ordinate (y) of each center of gravity was then multiplied by 2. (3) Reconstruction of a placentone with 4 vertical corrected sections assembled by axial symmetry. B: Velocity plots for the 4 inlet velocities (50, 100, 200 and 300 mm.s^-1^). C: Mean wall shear stress (WSS) (dyn.cm^-2^) in the IVS as a function of the inlet velocity. D: For an abscissa (x = 1), WSS (dyn.cm-^2^) as a function of y (heigh in the IVS).

In the second model, a 2 cm vertical cross-section image from the chorionic plate to the basal plate of a third-trimester normal placenta was used to represent the villous tree inside the placentone as a realistic, inhomogeneous, anisotropic and rigid medium. The images were scanned and then digitized using Adobe Illustrator CS6 software. In order to obtain a more physiological model, we applied a correction for the placental collapse that occurs after delivery (Fig 2A2). This correction consisted of determining the x- and y-coordinates of the centers of gravity of each solid element in the image and subsequently stretching these solid elements by a factor of 2 in the y-direction, which avoided modification of the size and shape of the solid elements. The digitized images were imported, copied and reflected to create the final geometry (Fig 2A3), and this geometry was enclosed in a 4x4 cm square chamber. The spiral artery entering the IVS was modeled by a 2 mm-wide inlet positioned at the center of the bottom surface of the chamber [5] while two identical 2 mm-wide outlets along the bottom surface represented the decidual veins. Image J software (http://imagej.nih.gov/ij/index.html) was used for image processing and analysis including gray scale conversion (conversion of RGB images into 8-bit images), threshold segmentation, and automatic conversion of images into binary images. The total porosity ϕ was calculated as the sum of the areas of all the pores divided by the total area of the field, expressed as a percentage. No slip boundary conditions were applied to the walls of the domain, and flow information was reflected across the interfaces between images. The flow field of maternal blood in this model of the IVS was obtained by solving the Navier-Stokes equations.

### Model 3: Three-dimensional modeling of a terminal villus in the intervillous space

**Fig 3:**
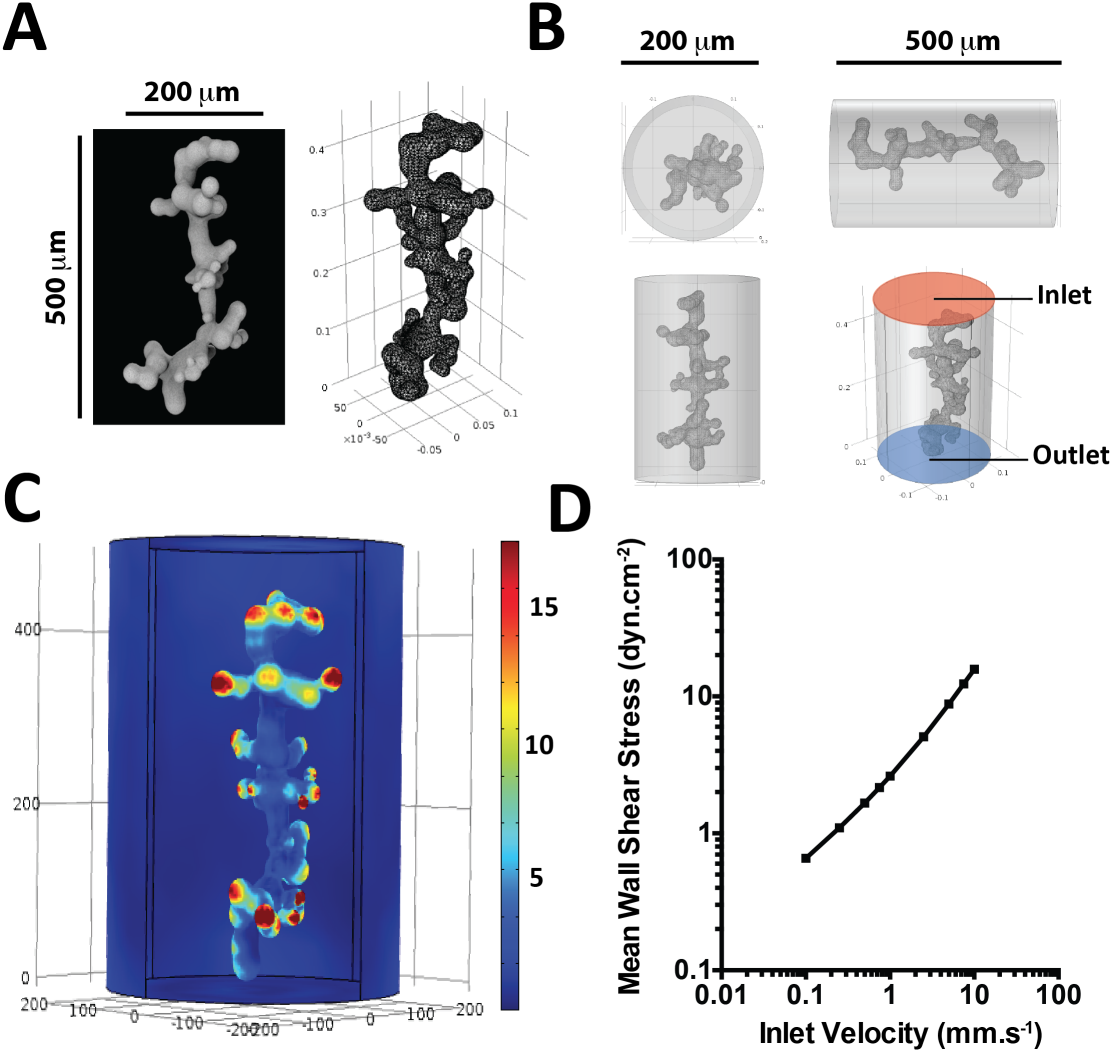
3D modeling and numerical simulation of the maternal circulation around a terminal villosity. A: 3D reconstruction of a terminal villosity from a scanning electron microscopic image of a third-trimester terminal villosity. B: 3D geometry built in COMSOL Multiphysics and used in the simulation; the terminal villosity is positioned inside a cylinder with a 200 μm diameter and 500 μm height. C: 3D plot of the wall shear stress exerted on the surface of the terminal villosity (inlet velocity = 1mm.s^-1^). D: 3D mean wall shear stress exerted on the surface of the terminal villosity as a function of input velocity in the cylinder.

Starting with a scanning electron microscopy image, AutoCAD 18.0 software (Autodesk, Inc. San Rafael, CA 94903) was used to reconstruct in 3D the terminal villus in the center of the IVS (Fig 3A). The 3D volume was imported into COMSOL Multiphysics 4.4 software (COMSOL, Inc. MA 01803 USA) and positioned within a cylinder 200 μm in diameter and 500 μm in height (Fig 3B). In this model, the inlet was represented as the top of the cylinder and the outlet as the bottom of the cylinder. No-slip conditions were applied to the surface of the villus, and slip boundary conditions were applied to the side walls of the cylinder to model the larger fluid volume within which the villus would be present *in vivo*. The Navier-Stokes equations were solved to determine the flow field and the WSS on the villus surface.

### Computational Fluid Dynamic Simulations

Computations of the flow fields in all models were performed using the CFD module of the commercial finite element code COMSOL Multiphysics 4.4. In all simulations, flow into the spiral artery is assumed to be steady. In the central cavity of Model 1 and in Models 2 and 3, the flow field is obtained by solving the equations of conservation of mass and linear momentum (the Navier-Stokes equations) as follows:

Mass:

∇.v=0

Momentum:

$$\rho \left[\frac{\partial v}{\partial t}+\nabla v.v\right]=-\nabla p+\;\mu \nabla^{2}v$$

where v is the flow velocity vector, ρ is the fluid density, p is the pressure, μis the dynamic viscosity, and ∇ is the del operator.

Within the porous media portion of Model 1, we assume that the flow is governed by the Brinkman equation as follows:

$$\frac{\rho }{\varphi }\;\left[\;\frac{\partial v}{\partial t}\;+\;\frac{\partial v.v}{\varphi }\right]=\;-\nabla p+\;\frac{\mu }{\varphi }\nabla^{2}v-\;\frac{\mu v}{k}-\;\frac{F\rho \left|v\right|v}{\sqrt{k}}$$

where F is the Forchheimer coefficient reflecting the influence of the inertia terms at the pore scale, ϕ is the porosity (taken as 0.5 [6]), and k is the permeability (10^−8^ m²). The permeability value is an order-of-magnitude estimate based on a typical pressure drop of the order of 1 mmHg in *ex vivo* perfusion experiments in an isolated placental lobule [5].

Maternal blood was modeled as an incompressible shear-thinning non-Newtonian fluid. The Carreau model was used to describe the dynamic viscosity μ as a function of the strain rate $\dot{\gamma }$ as follows:

$$\mu \left(\dot{\gamma }\right)=\;\mu_{\infty }+(\mu_{0}-\mu_{\infty })\;.{\left[1+{\left(\lambda \dot{\gamma }\right)}^{2}\right]}^{\frac{n-1}{2}}$$

where μ_∞_ and μ_0_ are the values of the dynamic viscosity at infinite and zero shear rate, respectively, λ is the time constant associated with the dynamic viscosity changes with shear rate, and n is the power law index. The Carreau model values adopted are as follows: μ_∞_ = 0.0035 kg m^-1^s^-1^ (which corresponds to the value of dynamic viscosity used in the Newtonian flow simulations), μ_0_ = 0.25 kg m^-1^s^-1^, λ = 25 s, and n = 0.25 [7].

The hemodynamic parameters applied in the 2D models (Models 1 and 2) correspond to the peak systolic velocities measured by Doppler ultrasound in the spiral arteries in third-trimester pregnancies [8–9] or obtained from mathematical models [10]. Four values of inlet velocities were applied (50, 100, 200, 300 mm.s^-1^). The hemodynamic parameters applied in the 3D model (Model 3) correspond (1) to the blood velocities measured by MRI in the center of the IVS, and (2) to the values reported in the scientific literature [10]. These velocities are much lower than those measured at the entrance of the IVS [8]. In both the 2D and 3D models, the outlet pressure in the simulations was set to 0 mmHg. A mesh independence study was performed to ensure sufficient fineness of the computational meshes used in the simulations. The typical size of the finite element mesh used was 0.141 mm^2^ (41 371 elements) in Model 1 and 0.043 mm^2^ (1 402 207 elements) in Model 2. In the 3D model, the typical size of the finite element mesh used was 64x10^3^ μm^3^ (176 907 elements).

### Dynamic placental MRI

The *in vivo* dynamic MRI measurement was part of a clinical trial (PLACENTIMAGE Study, clinical trials.gov Identifier: NCT01092949). The data from 5 pregnant women who underwent prenatal MRI for fetal malformation without placental pathology between 30+4 and 34 weeks of gestation were used for these analyses. Placental MRI was performed using a 1.5 Tesla unit (Sigma, GE, Milwaukee, Wis) with a phased-array abdominal coil. The MRI protocol included anatomical sequences of the placenta in the three orthogonal planes (FIESTA 2D sequence: TR = 3.65; TE = 1.6; Matrix: 512x512; FA: 65°; Section thickness: 6 mm). We performed a multiphase FSPGR sequence (TR: 2.2; TE0.9; Matrix: 256x256; FA: 15°; Section thickness: 5 mm every 5 mm, 20 slices, pixels: 2.2x1.1mm^2^, voxels: 2.2x1.1x5 mm^3^) after injection of gadolinium chelate (Dotarem^®^, Guerbet, France, 0.05 mmol.kg^-1^). The temporal resolution was 4±0.1 s and was recalculated for each exam. The maternal blood velocity in the IVS was calculated between each acquisition by dividing the progression of the contrast medium by the temporal resolution Δt. The measurements were performed with clinical software (Advantage Workstation 4.6 Ver. 1.4—GE Healthcare) on axial sections, perpendicular to the axis of the body. The front of progression of the contrast-enhanced maternal blood appears as a strong hypersignal and is easily distinguishable from the non-enhanced placenta. After a zoom on the region of interest, the progression of the contrast-enhanced maternal blood was measured:

- At every Δt,
- From exactly the same voxel coordinates (x, y, z) of the basal plate,
- In a perpendicular direction between the basal plate and the chorionic plate,
- To the limit of progression of the contrast-enhanced maternal blood.

By subtraction, we calculated the segment length enhanced by contrast between each time interval Δt. Each length was divided by Δt to obtain the average velocity of maternal blood in the placentone. Three cotyledons were routinely analyzed for each placenta.

### Human trophoblastic primary cell culture

**Fig 4:**
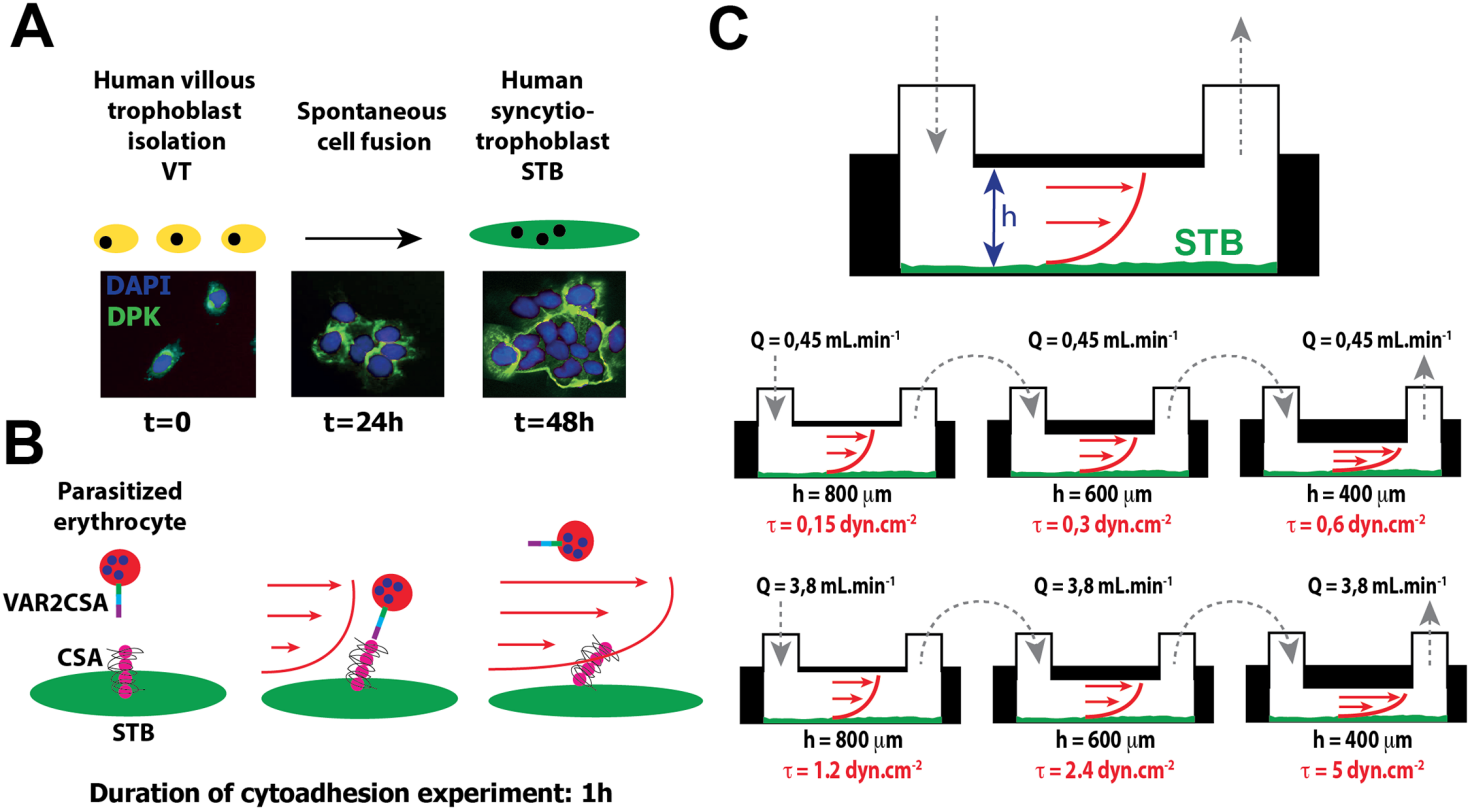
Experimental device for applying increasing levels of wall shear stress to the human syncytiotrophoblast in primary culture. A: Human syncytiotrophoblasts were obtained by spontaneous fusion of villous cytotrophoblasts (VT) after 48 hours of culture. VT and STBs were fixed and immunostained for desmoplakin (DPK, 5 μg.mL^-1^, *green*), and nuclei were counterstained with 4’,6-diamidino-2-phenylindole (DAPI, *blue*). B: STBs were exposed to steady unidirectional laminar shear stress with the culture medium containing human erythrocytes infected by *Plasmodium falciparum* (hematocrit: 1%, parasitemia: 2%) for one hour. Channel slides (sticky-slides Luer I 0.4, Luer I 0.6, Luer I 0.8, Ibidi^®^ GmbH Martinsreid, Germany) with increasing heights (h = 400/600/800 μm), arranged in series and connected to a pump system (Ibidi^®^ GmbH Martinsreid, Germany) generating flow rates of 0.45 mL.min^-1^ and 3.8 mL.min^-1^, were used to apply a increasing levels of wall shear stress (0.15, 0.30, 0.60, 1.2, 2.4 and 5 dyn.cm^-2^). C: Infections with the human malaria parasite *Plasmodium falciparum* during pregnancy lead to cytoadhesion of parasitized erythrocytes in the intervillous space. Cytoadherence is conferred by the specific interaction of the parasite-encoded adhesin VAR2CSA with chondroitin-4-sulfate A (CSA) present on human syncytiotrophoblast proteoglycans.

Third-trimester placentas were obtained immediately after planned cesarean section from healthy mothers who gave birth at 37–39 weeks of gestation. Cytotrophoblasts were isolated as previously described [11]. After sequential 0.20% trypsin (Difco Laboratories, Detroit, MI) and DNase I (Sigma, Saint-Quentin Fallavier, France) digestion followed by Percoll gradient centrifugation [12], the cytotrophoblasts were diluted to a final density of 10^6^ cells in 1 mL of Dulbecco’s modified Eagle’s medium (Gibco, Life Technologies) containing 10% fetal calf serum (Laboratories PAA), 2 mM glutamine, 100 IU.mL^-1^ of penicillin/streptomycin (Gibco, Life Technologies) and plated at 150,000 cells.cm^-2^ on microslides (Ibitreat Ibidi GmbH, Martinsreid, Germany) and incubated at 37°C in 10% CO_2_. STBs were obtained by fusion of villous cytotrophoblasts after 48 hours of culture (Fig 4A).

### *Plasmodium falciparum* culture and selection

Erythrocytes infected by *P*. *falciparum* (FCR3 laboratory strain) presenting a “CSA” binding phenotype were obtained by selection pressure from the FCR3 strain incubated in the presence of the CSA receptor, as previously described [13]. After 1 hour of incubation with the receptor, the infected erythrocytes (IEs) were washed gently with the culture medium. Only, the parasites expressing the VAR2CSA protein were linked to the receptor (Fig 3D). The rest of the parasite population was removed by washing. The FCR3-selected and non-selected “CSA” lines were then cultured for 6–8 cycles to generate sufficient parasite density [14]. Parasites were maintained in human erythrocytes (O+/-, blood bank, EFS PARIS, France). The culture medium consisted of RPMI 1640 complemented with 25 mM Hepes (Gibco, Life Technologies), 2 mM L-glutamine (Gibco, Fisher—Scientific Invitrogen), supplemented with 0.5% albuMAX II (Gibco, Life Technologies) and 2% of human AB serum (Laboratories PAA). For binding to STB, mature stages from FCR3-selected and non-selected “CSA” lines were enriched to 0.5–2% parasitemia by Percoll gradient separation. The mature stages were harvested and the hematocrit was adjusted to 5% with human erythrocytes. A control group was prepared by pre-incubation of the FCR3-selected “CSA” line with 10 μg.mL^-1^ soluble CSA for 1 hour at room temperature, validating the CSA-binding phenotype of the FCR3-selected “CSA” strain [15].

### WSS experiments

After 48 hours of culture, the syncytiae were rinsed with PBS 1X, and the microslides were placed in a parallel plate flow chamber for dynamic cell culture. The STB was exposed to steady unidirectional laminar WSS with the complemented RPMI 1640 containing the human erythrocytes for one hour (parasitemia: 2%, hematocrit: 1%). Sticky slides (Luer I 0.4, Luer I 0.6, Luer I 0.8, Ibidi GmbH Martinsreid, Germany) arranged in series and connected to a pump system (Ibidi GmbH Martinsreid, Germany) generating two flow rates of 0.45 mL.min^-1^ and 3.8 mL.min^-1^ were used to apply an increasing range of WSS (0.15, 0.30, 0.60, 1.2, 2.4, and 5 dyn.cm^-2^. With this approach the total number of IEs flowing through the chambers was constant for all hydrodynamic conditions.

### Immunocytochemistry

**Fig 5:**
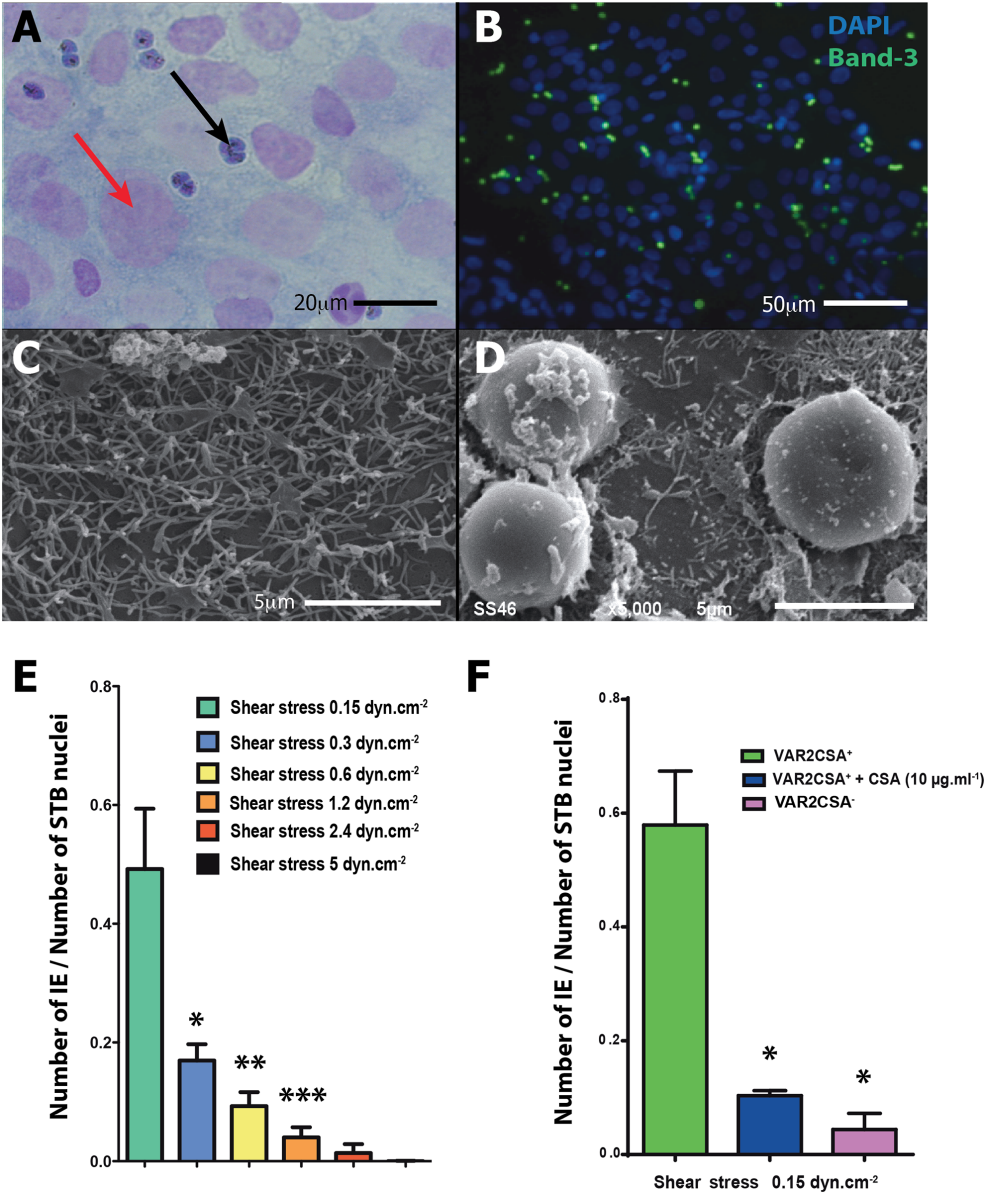
Cytoadhesion experiments with infected erythrocytes (IEs) under flow conditions with increasing levels of wall shear stress. A: Giemsa staining of IEs adherent to the STB after 1 hour under flow conditions (0.15 dyn.cm^-2^). Black arrow: IEs, red arrow: STB nucleus. B: IEs adherent to the STB, after 1 hour under flow conditions (0.15 dyn.cm^-2^). The plasma membrane of the adherent IE is immunolabeled with a monoclonal anti-human Band 3 (Clone BIII-136 B 9277 Sigma 0.1μg.ml^-1^) antibody. STB nuclei are labeled with DAPI. C: Scanning electron micrograph of the STB after 1 hour under flow conditions (0.15 dyn.cm^-2^) without erythrocytes. D: Scanning electron micrograph of the STB after 1 hour under flow conditions (0.15 dyn.cm^-2^) with IEs expressing VAR2CSA (VAR2CSA^+^). E: cytoadhesion quantification of IEs after 1 hour under flow conditions with increasing levels of wall shear stress (mean±SEM). * significant difference (p<0.05) compared with 0.15 dyn.cm^-2^. ** significant difference (p<0.05) compared with 0.60 dyn.cm^-2^. *** significant difference (p<0.05) compared with 1.2 dyn.cm^-2^. F: Comparison of cytoadhesion after 1 hour under flow conditions (0.15 dyn.cm^-2^) between IEs expressing VAR2CSA (IE VAR2CSA^+^), IEs VAR2CSA^+^ pre-incubated with soluble chondroitin-4-sulfate A (CSA 10 μg.mL^-1^) and IEs VAR2CSA^-^. * significant difference (p<0.05) compared with IEs VAR2CSA^+^.

After WSS application, the cells were carefully rinsed with cold PBS 1X and fixed with 4% paraformaldehyde (Sigma-Aldrich, Saint-Quentin, France) at 20°C for 20 min. The cells were incubated with 1% BSA in PBS for 60 min at room temperature. To detect the plasma membrane of the adherent erythrocytes, a mouse monoclonal anti-human Band 3 antibody (0.1 μg/mL; Clone BIII-136 B 9277 from Sigma), was applied overnight at 4°C, followed by an Alexa 488-labeled donkey anti-mouse antibody (Molecular Probes, Inc., Eugene, OR, 1/400) for 60 min at room temperature in the dark. STB nuclei and parasites were labeled with 6-diamidino-2-phenylindole (Fig 5B). The controls, obtained by excluding the primary antibody or applying the nonspecific IgG of the same isotype, were all negative. IEs and STB nuclei were counted in 10 fields per slide and per WSS regime to calculate the IE/STB nuclei ratio.

### Surface electron microscopy

Each sample was fixed with 2.5% glutaraldehyde (Sigma-Aldrich, Saint-Quentin, France) in PBS 1X at 20°C for 45 min. Post fixation was carried out using a 1% osmium tetroxide solution (Sigma-Aldrich) for 45 min at room temperature and followed by dehydration through graded ethanol series. Cells were critical point dried in hexamethyldisilazane (Sigma-Aldrich). For scanning electron microscopy, samples were mounted on specimen stubs and gold covered (1–2 nm) by cathodic spraying (Fine coat ion sputter JFC-1100, Jeol S.A., Tokyo, Japan) and examined at 10 kV using a JEOL electron microscope (JSM-6510LV, JEOL S.A., Tokyo, Japan).

## Results

### 2D computational fluid dynamic simulations

The results of the 2D computational fluid mechanical simulations (Models 1 and 2) demonstrated that blood velocities and WSS values in the IVS are highly heterogeneous spatially. In the porous media of Model 1, the mean fluid velocities in the IVS are lower than 1 mm.s^-1^ for inlet velocities lower than 200 mm.s^-1^ (Fig 1C). For points within the placentone that are 2 cm or more above the flow inlet, the WSS is smaller than 1 dyn.cm^-2^ (Fig 1D). In the more realistic case of Model 2 (Fig 2), inlet flow velocities of 50, 100, 200, 300 mm.s^-1^ are associated with mean WSS values in the IVS of 0.6±0.7, 1.1±1.5, 2.4±3.0 and 3.7±5.0 dyn.cm^-2^, respectively (Fig 2C), and the corresponding mean velocities in the IVS are 1.3±2.5, 2.7±5.1, 5.6±10.6, 8.8±16.6 mm.s^-1^, respectively (Fig 2B). The closer the villi are to the chorionic plate (fetal side), the lower is the WSS. This effect is more quantitatively depicted in Fig 2D which shows how WSS varies with the distance from the basal plate (y-direction) for a given x-position.

To demonstrate the robustness of the simulation results, we conducted an extensive parametric study by varying both the diameter of the input (spiral artery) from 1.5 mm to 2.5 mm (in increments of 0.025 mm) and the input flow velocity from 5 to 30 cm.s^-1^. The parametric study demonstrated that in Model 1, the WSS calculated at point A (where the WSS is maximum) varies from 2.4 to 3.7 dyn.cm^-2^ for a 30 cm.s^-1^ input velocity and from 0.3 to 0.6 dyn.cm^-2^ for a 5 cm.s^-1^ input velocity. The WSS calculated at point D (where the WSS is minimum) varies from 0.1 to 0.2 dyn.cm^-2^ for an input velocity of 30 cm.s^-1^ and from 0.01 to 0.02 dyn.cm^-2^ for an input velocity of 5 cm.s^-1^.

### 3D computational fluid dynamic simulations

For the inlet velocities of 0.1, 1 and 10 mm.s^-1^, the pressure drop between the inlet and the outlet in the 3D model is 0.18, 0.66 and 3.57 Pa, respectively. The WSS exerted on the external surface of the STB is spatially very heterogeneous (Fig 3C). Although the maternal blood velocities in the IVS cannot be precisely measured, they are estimated to range from 0.1 to 1 mm.s^-1^. According to our model, the mean WSS exerted on the surface of a terminal villus for this range of velocities is 0.5±0.2 to 2.3±1.1 dyn.cm^-2^ (Fig 3D).

### Erythrocyte cytoadhesion

The analysis using optical microscopy, immunofluorescence and SEM confirmed the presence of adherent erythrocytes on the surface of the STB after one hour of flow (Fig 5A). All adherent erythrocytes showed positive Giemsa staining under the optical microscope and DAPI staining in fluorescence (Fig 5B), which demonstrates that only erythrocytes infected with *Plasmodium falciparum* adhere to the STB. In the presence of soluble chondroitin-4-sulfate A (CSA) protein in the circulating medium, the cytoadhesion of the IEs was dramatically reduced (Fig 5F). These results suggest that the cytoadhesion is due to from the interaction of the parasite's proteins expressed on the erythrocyte's surface with the CSA on the STB. The experimental setup was designed so that during the same experiment the erythrocytes progressively underwent increasing WSS ranging from 0.15 to 0.6 dyn.cm^-2^ and from 1.2 to 5 dyn.cm^-2^. The number of adherent IEs after one hour was significantly lower at 0.3 dyn.cm^-2^ than at 0.15 dyn.cm^-2^. The same significant decrease was observed at each step of increased WSS. Beyond 5 dyn.cm^-2^ there was no observed cytoadhesion of IEs in one hour of flow (Fig 5E).

### Dynamic placental MRI

**Fig 6:**
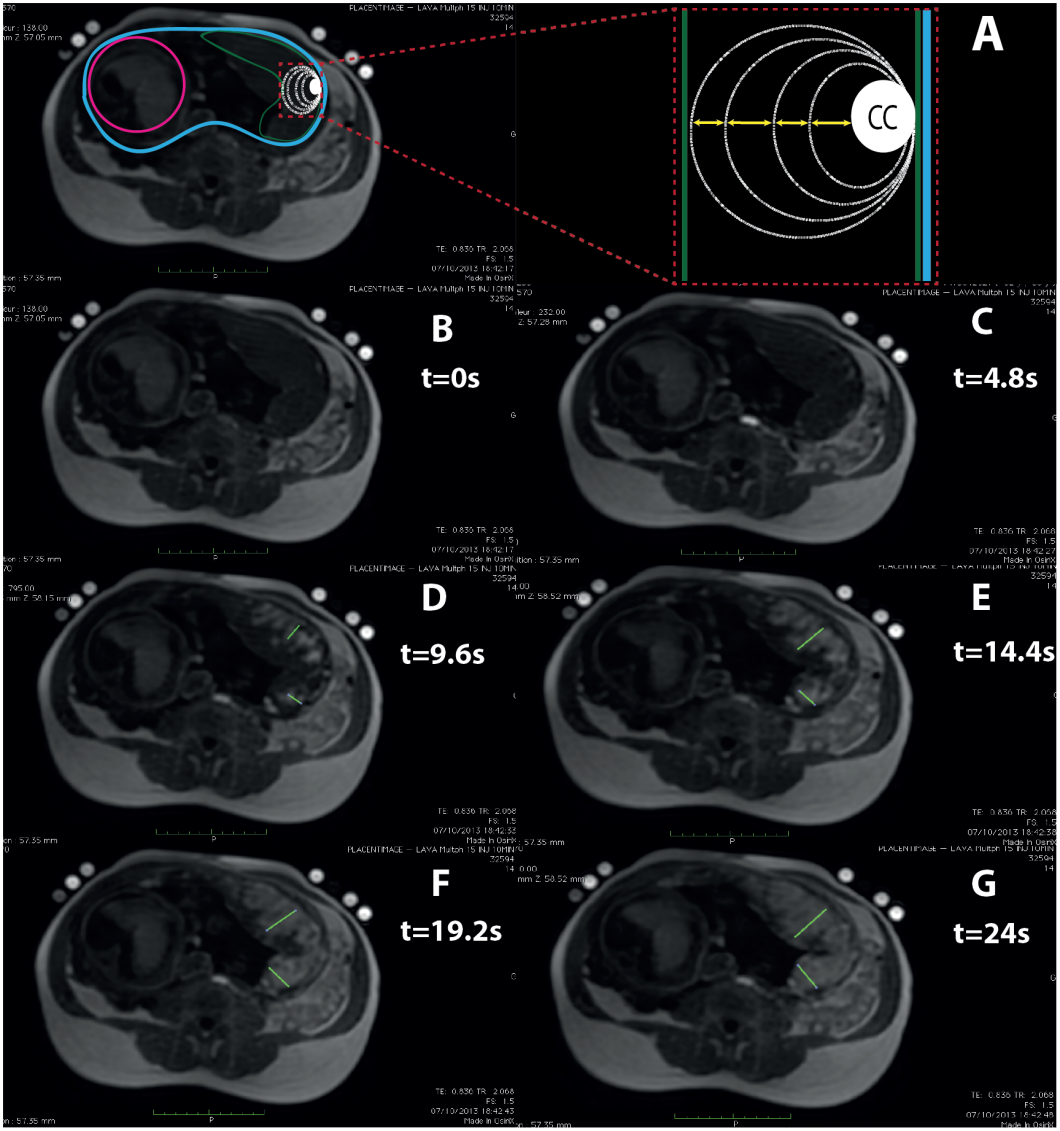
Dynamic placental MRI. A: Placenta (green line), uterus (blue line), fetus (pink line). The intraplacental progression of the contrast-enhanced maternal blood in the IVS was measured (yellow arrows) every Δt in a perpendicular direction between the basal plate and the chorionic plate. B-G: Progression of the gadolinium is measured every 4.8s, from the first MRI picture with gadolinium in the placenta (Fig 6D) in order to avoid calculating the velocity of the maternal blood in the central cavity (CC) of the placentone. The mean flow velocity of maternal blood measured in the IVS is 0.94±0.14 mm.s^-1^.

Determination of average velocities of maternal blood progression in the IVS by MRI allowed us to use and more accurately analyze the simulation results. In placental perfusion MRI, the intraplacental progression of the gadolinium corresponds to the progression of the maternal blood in the IVS. On the first MRI image with gadolinium in the placenta (Fig 6D), the average distance between the basal plate and the gadolinium progression front was 12.2±1.1 mm. This rapid contrast enhancement corresponds to the jets of maternal blood from the spiral artery into the central cavity (CC) of the IVS. The progression of the gadolinium was measured from the first MRI picture with gadolinium in the placenta (Fig 6D) in order to avoid calculating the velocity of the maternal blood in the central cavity of the IVS. The mean flow velocity of maternal blood in the IVS was 0.94±0.14 mm.s^-1^.

## Discussion

The primary objective of our study was to determine the WSS exerted by the maternal blood flow on the STB in the human placenta during the third trimester of pregnancy. We began by performing computational fluid dynamic simulations. To determine the WSS values on the scale of the placentone, we developed two numerical models of the placentone in 2D: (1) a homogeneous porous medium within which flow is governed by the Brinkman equations, and (2) a more realistic medium reconstructed from histological sections of human placenta. To evaluate the WSS exerted on the STB on the scale of the villi, we also developed a 3D model of a terminal villus. The two main results of the numerical simulations are that (1) the WSS exerted on the STB in human placenta is low compared with the rest of the vascular system and (2) the WSS values vary greatly across the IVS and the chorionic villus. These results can be explained structurally: (1) the IVS is a highly asymmetric open vascular space unlike a branched vascular network, and (2) the chorionic villus has a complex irregular convex surface, unlike the inner wall of the blood vessels, which is cylindrical, regular and concave in shape.

For the 2D models, we used inlet velocities measured by pulsed Doppler ultrasound at the entrance of the IVS and published in literature. Kurjak et al measured the maternal blood velocity in the spiral arteries [8]. Between 37 and 42 weeks of gestation the average peak systolic velocity and end-diastolic velocity were 30 and 19 cm.s^-1^, respectively. Collins et al^9^ used ultrasound to measure the velocity of blood in the maternal spiral arteries as it flows into the IVS, starting at the beginning of the second trimester. They recorded a drop in velocity from a mean of 54 to 20 cm.s^-1^ at 34 weeks. Extrapolating their results to 40 weeks, they calculated a velocity of 10 cm.s^-1^, which is that predicted by Burton’s mathematical model [10]. Because of these differences in measurements and estimations, we studied inlet velocities ranging from 5 to 30 cm.s^-1^.

For the 3D model, the velocities used correspond to the velocities of the maternal blood inside the IVS. These velocities have been estimated^5^, but to our knowledge they have not been measured *in vivo* in the human placenta. Therefore, to determine these values, we measured *in vivo* the gadolinium velocity in the IVS during MRI performed in the third trimester of pregnancy. The velocities in the intervillous space were ≈1 mm.s^-1^. Considering the blood velocities within the IVS calculated with dynamic MRI, our 3D model predicts a WSS of 0.5 to 2.3 dyn.cm^-2^ (0.05 to 0.2 Pa).

Several authors have identified the placental IVS as a porous medium [16–17]. In a homogeneous and isotropic porous medium completely filled with an incompressible fluid, where blood flow circulates at a low velocity (Reynolds number on the order of 1), the pressure gradients are proportional to the flow velocity in the pores (Hagen-Poiseuille law applied to each pore). The permeability (k) is a measure of the ability of the porous medium to allow fluids to pass through it. The permeability of a porous medium is dependent on the overall porosity, the specific area and the geometric characteristics of the pores. In our Model 1, the permeability is considered to be uniform and identical throughout the surface of placentone. We conducted a parametric study by varying both the permeability k from 10^−6^ to 10^−10^ m^2^ and the input flow velocity from 5 to 30 cm.s^-1^. For k = 10^−6^ m^2^, the average velocities in the IVS (surface 2) were 3.1, 10.1, 31.9 and 60.9 mm.s^-1^ as the input flow velocity was progressively increase. For k = 10^-7^m^2^, the corresponding average velocities were respectively 0.2, 0.8, 7.7 and 23.8 mm.s^-1^. Compared with the results obtained by MRI, these velocities calculated in the IVS were high, at both 10^−6^ and 10^−7^ m^2^ permeability values. We calculated the permeability k for Model 2 using the Darcy equation:

$$k=\frac{\mu .v.L}{\Delta P}$$

where μ is the blood dynamic viscosity (3.5.10^−3^ kg.m^-1^.s^-1^), v is the inlet velocity, L is the length of the placentone (4 cm) and ΔP is the pressure drop between inlet and outlet. The permeability value calculated is 10^−8^ m^2^. For these reasons we used the value of k = 10^−8^ m^2^ for the Brinkman equations in Model 1.

The 2D model developed based on histological sections (Model 2) circumvents the need to determine the permeability of the porous medium and the use of the Brinkman equations. Rather, the Navier-Stokes equations are directly solved within the flow domain. *In vivo*, the placental thickness in the third trimester of pregnancy measured by ultrasound [18] or MRI is approximately 4 cm. *Ex vivo*, the vertical histological sections from the basal plate to the chorionic plate (Fig 2A) measure 2 cm. At the time of the placental expulsion (afterbirth), the pressure drop in the IVS and the leakage of the maternal blood out of the IVS produce a collapse of the placenta onto itself. We have corrected for the vertical component of this collapse. We consider the surface area of the basal plate of the placenta to be similar after delivery to that *in vivo*. However, the average height of the placenta measured *in vivo* (4 cm) does not correspond to postnatal values (2 cm). Therefore, the correction was only performed in the y direction and not in the x direction. The placental collapse correction is a critical step for the construction of the model because it modifies the permeability of the medium by changing the geometric characteristics such as porosity and specific pore surface areas. The mean velocities in the IVS and the mean WSS exerted on the STB are affected by the correction of placental collapse. The estimated ratio of the volume of the villi per volume of placenta at term during a normal pregnancy is 43%[19], which is equivalent to 57% porosity, and corresponds approximately to our model with porous media. According to Cherniavsky et al, the placentone exhibits a trade-off between flow resistance and uptake capacity^5^. A higher density of villous material (lower porosity) provides a larger surface area for uptake but also higher flow resistance and hence lower perfusion. With a mathematical model describing flow of maternal blood in the placentone they have calculated an optimal volume fraction of 0.3 (porosity = 0.7, which corresponds to our realistic model), which maximizes the absolute net uptake rate of a representative solute.

The current numerical modeling has several limitations. First, the simulations did not take into account all the fluid-structure interactions. For the 2D/3D simulations the rigidity of the chorionic villi was considered infinite, but *in vivo* the structure can bend and/or deform under the influence of maternal blood flow. Fetal circulation causes pulsatile movements and cyclical variations in the rigidity of chorionic villi that we did not take into account.

The simulations were performed using steady (non-pulsatile) flow. The flow regime in the spiral arteries remains pulsatile until 20 weeks of gestation. In the third trimester, most inflow into the IVS is not pulsatile [9]. Moreover, the cavities in the center of the cotyledon dampen the pulsatility, which makes the circulating flow between the chorionic villi rather steady. The 3D geometry of the placentone *in vivo* is not particularly well described. We considered that for modeling the entire placentone, there are fewer approximations in 2D. So we carried out modeling and simulations in 2D for the entire placentone and in 3D for the terminal villus.

The simulations using the 2D models show that a significant portion of the flow circumvents the IVS and flows straight out. The distribution of flows in the IVS depends on the position of the output ports. Classically, for mathematical models of intervillous blood flow in the human placentone, the outputs are placed in the basal plate. However, the draining veins of the IVS are classically described in the basal plate and in the lateral septa, which separate the cotyledons [20]. We performed simulations with the two outputs placed on the lateral sides of the IVS, and the distribution of flows was deeper within the placentone (data not shown), but the values of WSS were not significantly different.

The second part of our work was to ascertain that the *in silico* fluid dynamic results are consistent with physiological observations. To this end, we evaluated the cytoadhesion of IEs to the STB *in vitro* under flow conditions at different WSS values. Susceptibility to pregnancy-associated malaria likely represents a combination of immunological and hormonal changes associated with pregnancy, combined with the unique ability of a subset of IEs to sequester in the placenta [21]. *In vivo*, IEs express the membrane protein VAR2CSA, which binds to chondroitin-4-sulfate A (CSA), a component of the proteoglycan matrix at the apical surface of the STB cell layer [22]. Our experiments with IEs demonstrate that the levels of WSS obtained by numerical simulations were able to reproduce this cytoadhesion. We hypothesized that increasing the flow rates and the WSS at the fluid-structure interface would have the effect of reducing cytoadhesion. We also postulated that physiological WSS is probably lower than the threshold beyond which there is no more cytoadhesion *in vitro*. In our experimental conditions, the results showed that there is no cytoadhesion at a WSS higher than 5 dyn.cm^-2^. We conclude that *in vivo*, in the areas where there is cytoadhesion between IEs expressing VAR2CSA and the STB, the WSS is likely to be less than 5 dyn.cm^-2^.

The mean values of WSS exerted on the STB in the IVS by maternal blood flow are quite low in comparison with the rest of the cardiovascular system [4]. However, small values, as well as small variations in WSS, can indeed be sensed and integrated by the cells. In the proximal tubule cells, the WSS (≈1.0 dyn.cm^-2^) creates a bending moment on the microvilli in which there is an organized central actin filament bundle. Although the predicted force on each microvillus is small, using a moment balance, Weinbaum et al showed a ~40-fold amplification of the force arising from the resisting moment of the central actin filament bundle at the base of the microvilli [23]. This amplification is sufficient to deform the anchoring filaments in the actin cortical web and initiate signaling through linker molecules such as ezrin [24]. As shown in Fig 5C, numerous microvilli are present on the surface of the STB in primary culture. The STB microvilli are supported by an underlying cytoskeleton consisting mainly of actin microfilaments [25–26–27]. Berryman et al have shown that ezrin is a major protein component of placental microvilli too, comprising ~5% of the total protein mass and present at about one quarter of the molar abundance of actin [28]. By homology, the mechanical forces exerted on the microvilli of the STB could have cellular effects via a mechanotransduction process.

## Conclusion

In spite of high maternal blood flow rates, the average WSS exerted on the chorionic villi is low (<5 dyn.cm^-2^). This study opens a new area of placental research that focuses on investigating the biological impact of WSS on human STB. During pregnancy, the STB, in addition to its endocrine and maternal-fetal exchange functions, exhibits “endothelial-like” functions such as nitric oxide production. We assume that the flow-dependent shear forces, as in the endothelium, may have an impact on the biological functions of the STB. The average WSS determined in this study (0.5±0.2 to 2.3±1.1 dyn.cm^-2^) could be applied in a controlled manner to STB primary cell culture as described in the present cytoadhesion experiments, and the cellular responses could be compared between flow conditions and static conditions.

## Abbreviations

STB: syncytiotrophoblast
CT: cytotrophoblast
IVS: intervillous space
CC: central cavity
WSS: wall shear stress
IE: infected erythrocyte
SD: standard deviation
CSA: chondroitin-4-sulfate A
MRI: magnetic resonance imaging
TRP: transient receptor potential
